# Clinical Features, Biochemical Parameters, and Treatment Adherence of Individuals Who Started the Treatment for Active Pulmonary Tuberculosis during the Pandemic Period

**DOI:** 10.3390/jcm12144843

**Published:** 2023-07-22

**Authors:** Amanda Caroline de Souza Sales, Larissa Araújo Lopes, Maria Caroliny dos Santos Vale, Mayara Ferreira Costa, João Victor de Souza Lima, João Gabriel Matos da Silva, Bruna Sthefanny da Cunha Ferreira, Victoria Alves do Nascimento, Saara Emanuele da Silva Flor, Elane Luiza Costa de Sousa, Bruna Katarine Bezerra Paz, Ricardo Amorim de Sousa Garcia, Eduardo Martins de Sousa, Alexsandro Ferreira dos Santos, Luís Cláudio Nascimento da Silva, Adrielle Zagmignan

**Affiliations:** 1 Laboratory of Microbial Pathogenesis, CEUMA University, São Luís 65075-120, MA, Brazil; 2 Postgraduate Program in Microbial Biology, CEUMA University, São Luís 65075-120, MA, Brazil; 3 Laboratory of Clinical Analysis (LABORCEUMA), CEUMA University, São Luís 65075-120, MA, Brazil; 4 Postgraduate Program in Health and Services Management, CEUMA University, São Luís 65075-120, MA, Brazil; 5 Laboratory of Immunology and Microbiology of Respiratory Infections, CEUMA University, São Luís 65075-120, MA, Brazil; 6 Departament of Nutrition, CEUMA University, São Luís 65075-120, MA, Brazil

**Keywords:** adverse drug reactions, biochemical markers, bacterial infections

## Abstract

This descriptive prospective study investigated the clinical features and treatment adherence of individuals who started the treatment for Pulmonary tuberculosis (TB) during the COVID-19 pandemic in São Luís. Thirty-six TB patients and thirty-five age/sex-matched individuals were recruited between January 2021 and January 2022. The clinical features, sociodemographic information, and serum were obtained at the diagnosis time. Adherence to treatment and adverse reactions were investigated monthly. The most common symptoms in TB patients were cough (91.6%) and fever (83.3%). All TB patients had elevated pre-therapy levels of CRP and reduced HDL: 88.9% presented hypocalcemia and 47.2% showed elevated ALP and GGT. TB patients showed higher levels of ALT, AST, ALP, GGT, CRP, amylase, and triglycerides than the comparison group (*p* < 0.05), while the calcium levels were reduced (*p* < 0.0001). TB patients with anti-SARS-CoV-2-IgG antibodies (seroprevalence of 66.7%) presented higher values of amylase and lower CRP levels (*p* < 0.05). Most patients (~70%) reported at least one adverse drug reaction, mainly pruritus and nausea. The treatment abandonment rate was 19.2%. In conclusion, TB patients showed elevated pre-therapy levels of CRP, low levels of HDL, and hypocalcemia. Liver and pancreatic functions were also compromised in several patients before the therapy. The treatment non-adherence rate observed was similar to other studies performed before the pandemic period.

## 1. Introduction

Tuberculosis (TB) is an infectious disease caused by *Mycobacterium tuberculosis* which remains one of the biggest threats to public health, especially for low- and middle-income countries [1,2,3]. In the last two decades, investments in TB control have decreased incidence and deaths [4]. In 2019, 10 million people developed TB worldwide, with more than 1.4 million deaths. However, COVID-19 displaced TB as the top cause of mortality among infectious diseases in 2020 [5].

The COVID-19 pandemic situation aggravated the sub-optimal global response to TB due to the substantial disruptions to health services and the worsening of poverty-related determinants [6]. Brazil, one of the thirty high-TB-burden countries, observed a reduction in TB incidence during the first two years of the COVID-19 pandemic (2020 and 2021). This panorama is associated with the restriction on TB diagnostic and treatment services, as well as less inclination and possibility to seek health care due to COVID-19 prevention and blocking strategies [6,7].

Among the strategies for the effective control of TB, early diagnosis and treatment stand out, and the delay in diagnosis due to the limitations of the tests and the similarity of symptoms with other respiratory diseases can contribute to the spread of TB [8]. The treatment of drug-sensitive TB in Brazil requires at least six months of directly observed therapy (DOT) in two regimens: an intensive 2-month phase with rifampicin, isoniazid, pyrazinamide, and ethambutol and then a 4-month continuation phase with isoniazid and rifampicin [9].

TB treatment is commonly associated with adverse drug reactions, which may contribute to poor adherence to treatment [10,11]. In many high-TB burden settings, around 15% or more of TB patients do not complete the treatment [12]. Incomplete adherence to treatment can lead to relapses, a worsening of clinical parameters, the development of drug resistance, as well as continuity of the transmission chain. All these factors negatively contribute to the health and economic well-being of patients, caregivers, and health systems [13]. 

Another issue associated with TB treatment is the high hepatotoxicity induced by the used drugs [14]. In this sense, investigating the biochemical parameters before anti-tuberculosis therapy becomes interesting, especially liver function. Indeed, some serum biomarkers have been indicated as useful to aid in diagnosis and prognosis in individuals with TB [15,16,17]. However, these serum biomarkers are not routinely tested for TB patients in São Luís, a Brazilian pre-Amazon city endemic for pulmonary TB [3].

In this context, this study investigated the pre-therapy clinical features and biochemical parameters of individuals who started the treatment for active pulmonary tuberculosis during the COVID-19 pandemic in São Luís. The prevalence of anti-SARS-CoV-2 antibodies in the patients was also investigated and correlated with the clinical findings. In addition, adherence to treatment and adverse reactions were investigated monthly.

## 2. Materials and Methods

### 2.1. Study Design

This descriptive prospective study aimed to investigate the clinical features and treatment adherence of individuals who started the treatment for TB. It was conducted with a convenience sample of patients recruited at a tertiary referral unit in São Luís, Brazil, between January 2021 and January 2022. The comparison group consisted of age- and sex-matched volunteers recruited at CEUMA University between September 2021 and January 2022.

### 2.2. Ethical Statements 

The study was conducted under Resolution 466/12 of the National Health Council of Brazil, Declaration of Helsinki II (2000). The sampling was conducted after approval by the Research Ethics Committee (CEP) of CEUMA University (Process number: 4.657.164). Written informed consent was obtained from all study participants.

### 2.3. Study Population and Data Collection

The active pulmonary TB patients (case group) were recruited at ‘Hospital Presidente Vargas’, a high-complexity hospital of reference for infectious diseases in São Luís. Convenience sampling was performed during January 2021 and January 2022. The patients were recruited before any treatment was started. At this point, blood collection was performed along with the collection of sociodemographic data (gender, age, marital status, education level, family information, and income), clinical features (diagnosis methods, exam results, medical conduct adopted, symptoms, comorbidities), and other characteristics through questionnaires and from the medical records. The adherence to treatment and possible side effects were monitored monthly by phone calls.

The inclusion criteria for the case group were adult patients (>18 years) diagnosed with active pulmonary TB (individuals with characteristic symptoms, suggestive radiography, positive sputum smear microscopy, culture and/or Xpert MTB/RIF assay) following the consensus statement from the Brazilian Thoracic Association [18]. The exclusion criteria were use of immunosuppressant drugs, co-infection with HIV, pregnancy, TB relapses, extrapulmonary TB. All enrolled patients signed the informed consent and correctly completed the structured questionnaire.

The comparison group consisted of age- and sex-matched volunteers with no previous diagnosis of TB and without chronic and parasitic manifestations. These individuals were recruited at CEUMA University between September 2021 and January 2022 and also signed the informed consent. Their sociodemographic data and behavioral characteristics were collected through questionnaires.

### 2.4. Blood Collection and Serum Analysis

The peripheral venous blood was drawn and collected from each individual at the time of enrollment. The samples were centrifuged at 1200× *g* for 10 min at 4 °C within 4 h of collection. The serum was transferred to an RNase/DNase-free tube and stored at −80 °C. The prevalence of anti-SARS-CoV-2 antibodies was investigated using the COVID-19 IgG/IgM Combo-ECO Diagnostics Rapid Test, following the manufacturer’s protocol. The liver function was evaluated with the measurements of Alanine aminotransferase (ALT), aspartate aminotransferase (AST), and γ-glutamyltransferase (GGT). C-reactive protein (CRP), uric acid, amylase, blood glucose, serum calcium, and lipid profile were also measured. All tests were performed in the biochemical analyzer LABMAX PLENNO–LABTEST. 

### 2.5. Assessment of Adherence to Anti-Tuberculosis Treatment

The treatment adherence was monitored monthly through a questionnaire (Table S1) completed with phone calls which assessed the possible changes in the treatment, non-probed self-reported adverse reactions, clinical evolution, and other health problems. The time from the beginning of standard treatment by the Brazilian Ministry of Health to discharge due to cure or end of treatment was considered. The outcome was monthly reported as adherent (those who adhere to the treatment) or non-adherent (those who did not adhere to the treatment). Those who could not be contacted were considered dropouts. The final indicator of adherence was also reported considering the full treatment. 

### 2.6. Statistical Analysis

GraphPad Prism 8.4.3 (San Diego, CA, USA) was used for data analysis. Categorical variables were analyzed using Chi-square (Χ^2^) test. Student’s *t*-test and Mann–Whitney test were applied for parametric and non-parametric variables, respectively. A value of *p* < 0.05 was considered statistically significant. Correlations were calculated using the Pearson coefficient (*ρ*) and classified as Very strong (*ρ* > 0.7), Moderated (0.6 ≤ *ρ* ≤ 0.7), Fair (0.3 ≤ *ρ* < 0.6), Poor (0.2 ≤ *ρ* < 0.3), and negligible (*ρ* < 0.2) [19].

## 3. Results

### 3.1. Analysis of Methods Used for Diagnostic of TB and Sociodemographic Characteristics of the Study Groups

**Figure 1:**
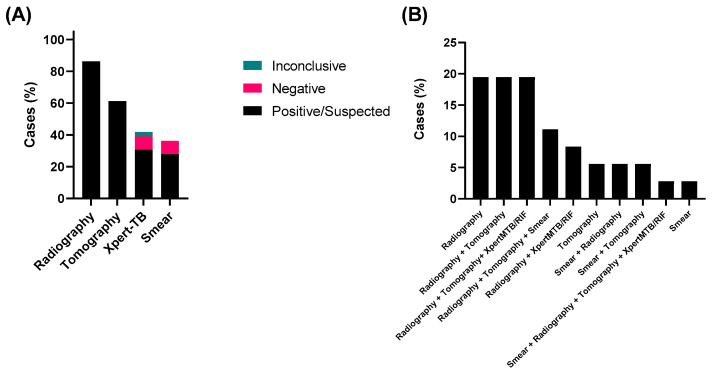
Methods applied in the diagnosis of active pulmonary TB. (**A**) Application of each method for investigation of active TB; (**B**) Methods used for starting the treatment against TB.

A total of seventy-one individuals were included in the study: thirty-six from the group with active pulmonary TB and thirty-five from the comparison group. Among the methods used to start the treatment against pulmonary TB in the studied hospital, radiography was the most performed (86.1%), followed by tomography (61.1%), Xpert-TB (30.6%), and smear (27.8%) (Figure 1A). In particular, the results of radiography alone combined with Tomography (“Radiography + Tomography”) or Tomography and XpertMTB/RIF (“Radiography + Tomography+ XpertMTB/RIF”) were used to start the treatment of most cases (19.44% for each). (Figure 1B). Sputum smear and XpertMTB/RIF (alone or in combination with other methods) were performed in 36.7% (positive for 27.8%) and 41.7% (positive for 30.55%) of the cases, respectively.

The TB group was mainly composed of men (69.4%) with a mean age of 34.9 years (SD = 12.5) and a predominance of age groups from 18 to 24 years old (22.2%) and 25 to 34 years old (30.6%). Regarding marital status, most of the individuals with TB were single (55.6%), and more than half had not finished high school (72.2%). They were predominantly auto-identified as ‘Pardo’ (mixed) (66.7%), followed by black (27.8%). For the majority (91.7%), the total monthly income of the family was up to three minimum wages (3300 Brazilian reais or approximately 600 USD). The same characteristics were observed in the comparison group, with a mean age of 34.7 (±12.6) years. Concerning the comparison group, most were single (68.6%), with complete high school (40.0%), auto-identified as ‘Pardo’ (60.0%), and with a family income of up to three minimum wages (62.9%) (Table 1).

Regarding other health characteristics (Table 2), previous contact with TB or leprosy individuals was reported by 50% and 11% of the patients, respectively. Most of them (83.3%) have the TB vaccine scar and did not undergo the tuberculin test (77.8%). Only a few patients reported comorbidity (16.8%) or other lung diseases (8.4%). The frequent use of alcohol and tobacco was informed by 47.2% and 30.6%, respectively.

### 3.2. Prevalence of Anti-SARS-CoV-2 Antibodies in Patients Recently Diagnosed with Active TB

Following this, the prevalence of anti-SARS-CoV-2 antibodies in patients with active TB was determined. Twenty-four individuals (67%) present anti-SARS-CoV-2 IgG antibodies. Anti-SARS-CoV-2 IgM-positive individuals were not detected. However, only 6 (25%) individuals from the IgG(+) group were sure about a previous infection by SARS-CoV-2 (16.7% of total TB patients), while 50% reported experiencing typical symptoms of COVID-19. In addition, 11 (45.8%) individuals claimed to have already taken a dose of a type of vaccine against SARS-CoV-2. In IgG(−) group, two (16.5%) individuals claimed to have already taken a dose of the vaccine against COVID-19, and 3 (25%) reported having experienced typical symptoms of COVID-19 (Table 3).

### 3.3. Clinical Manifestation of Pulmonary Tuberculosis 

The frequency of signs and symptoms of tuberculosis before treatment is represented in Table 4. The TB individuals had a median of four signs/symptoms. Cough was the most prevalent symptom (91.7%), followed by weight loss (88.9%), fever (83.3%), and weakness (58.3%). Only 27.78% and 22.2% related inappetence and hemoptysis, respectively. Although the signs and symptoms were more often reported by TB patients with Anti-SARS-CoV-2, a statistical difference was only observed for weakness (*p* = 0.0314) (Table 4). 

### 3.4. Measurement of Pre-Therapy Serum Biomarkers in Patients Recently Diagnosed with Active Pulmonary Tuberculosis 

The results of the measurement of pre-therapy serum biomarkers are shown in Table 5. All TB patients had elevated levels of CRP (>6 mg/dL) and low levels of HDL (M: <55 mg/dL; F: <65 mg/dL). Most TB patients (88.9%) presented hypocalcemia (<8.8 mg/dL), while hypercalcemia (>11 mg/dL) was detected in 5.6%. A considerable number of subjects with TB (47.2%) also presented elevated concentrations of ALP (>100 U/L) and GGT (M: >55 U/L; F: >38 U/L). High triglycerides (>120 mg/dL) and high amylase (>125 U/L) were seen in 22.2% and 13.9% of TB subjects, respectively.

**Figure 2:**
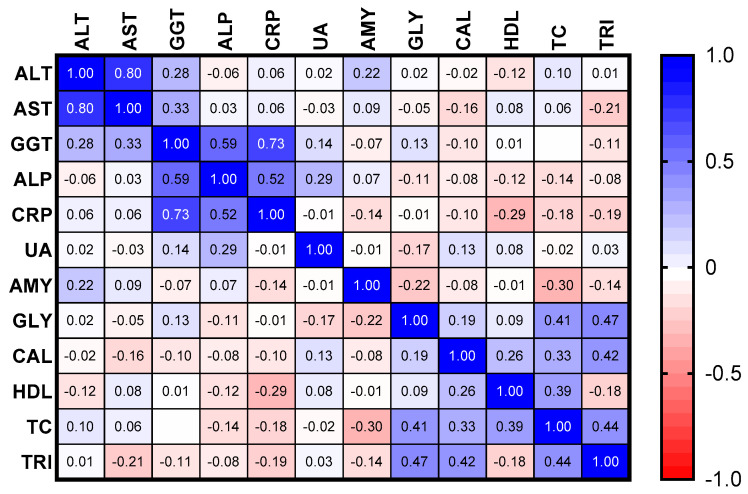
Correlation matrix of pre-therapy serum biomarkers in newly-diagnosed tuberculosis patients. ALT: Alanine aminotransferase, AST: aspartate aminotransferase, GGT: γ-glutamyltransferase, CRP: C-reactive protein, UA: uric acid, AMY: amylase, GLY: blood glucose, CAL: serum calcium, HDL: high-density lipoprotein cholesterol, TC: total cholesterol, TRI: triglycerides.

The correlations among the pre-therapy serum biomarkers in newly-diagnosed TB patients are represented in Figure 2. Positive strong correlations were observed for ALT and AST levels (*ρ =* 0.80) and CPR and GGT (*ρ =* 0.73), while the positive associations were classified as moderate for ALP with GGT (*ρ =* 0.59) and CRP (*ρ =* 0.52). Fair positive correlations were detected for AST with GGT (*ρ =* 0.33), total cholesterol with triglycerides (*ρ =* 0.44), HDL (*ρ =* 0.39) with Calcium (*ρ =* 0.33), triglycerides with glycemia (*ρ =* 0.47) and calcium (*ρ =* 0.42). Finally, positive poor correlations were detected for ALP with Uric acid (*ρ =* 0.29), GGT with ALT (*ρ =* 0.28), amylase with ALT (*ρ =* 0.22), and HDL with calcium (*ρ =* 0.26) while negative poor correlations were detected for amylase with total cholesterol (*ρ =* −0.30) and blood glucose (*ρ* = −0.22), CRP with HDL (*ρ* = −0.29), AST with triglycerides (*ρ* = −0.21) 

**Figure 3:**
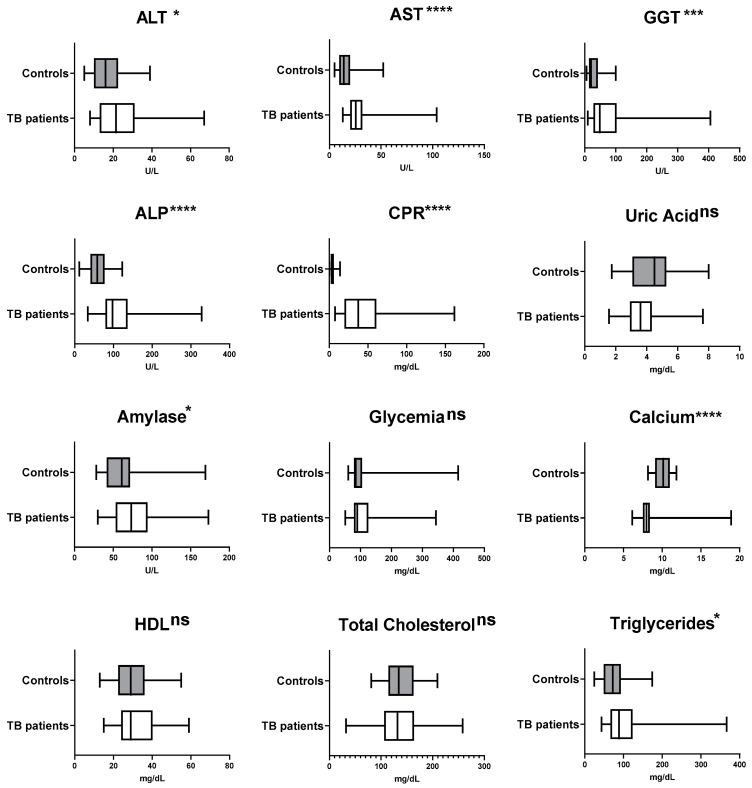
Comparison of median laboratory values for pre-therapy biochemical parameters in newly-diagnosed tuberculosis patients (*n* = 36) and comparison group (*n* = 35). Alanine Aminotransferase (ALT), Aspartate Aminotransferase (AST), and C-reactive Protein (CRP). * *p* < 0.05; *** *p* < 0.001; **** *p* < 0.0001. ns = no significant difference.

In addition, compared with the comparison group, patients with active pulmonary TB showed significant increases in ALT (*p* < 0.05), AST (*p* < 0.0001), ALP (*p* < 0.0001), GGT (*p* < 0.001), CRP (*p* < 0.0001), amylase (*p* < 0.05), and triglycerides (*p* < 0.05), while the levels of serum calcium were significantly reduced (*p* < 0.0001) (Figure 3). Regarding HDL, the levels in the comparison group were below the reference values, and statistical differences were not observed in relation to TB patients (*p* > 0.05).

**Figure 4:**
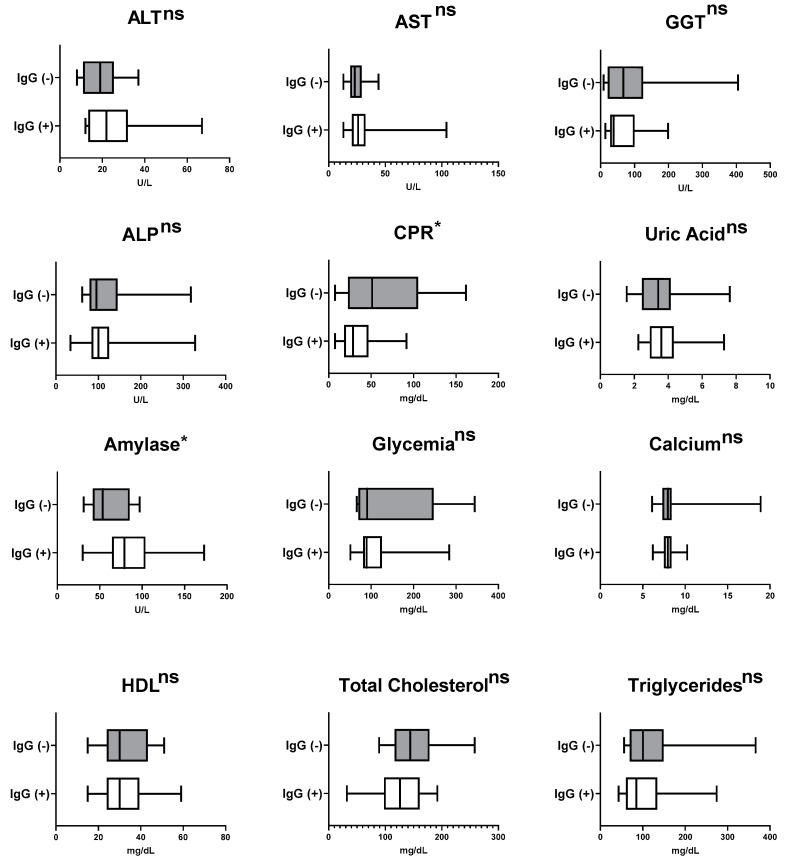
Comparison of median laboratory values for pre-therapy biochemical biomarkers in newly-diagnosed tuberculosis patients (*n* = 36) related to SARS-CoV-2 serology. * *p* < 0.05. ns = no significant difference.

Interestingly, those patients with active pulmonary TB who were positive for anti-SARS-CoV-2 IgG presented the lowest CRP levels (*p* < 0.05) and the highest values of amylase (*p* < 0.05), while no significant differences were observed for other serum biomarkers evaluated in this study (Figure 4). In patients with anti-SARS-CoV-2 IgG, CRP levels have fair positive associations with GGT (*ρ* > 0.58) and ALP (*ρ* > 0.48) and fair negative association with HDL (*ρ* > −0.34), while in the IgG, negative subjects show CRP levels with a very strong correlation with GGT (*ρ* > 0.78) and ALP (*ρ* > 0.64), moderate positive correlation with AST (*ρ* > 0.50), moderate negative correlation with triglycerides (*ρ* > −0.50), and fair negative association with HDL (*ρ* > −0.31). In relation to amylase, the association between this enzyme and GGT was fair for patients with or without anti-SARS-CoV-2 IgG; however, it was positive for IgG+ (*ρ* > 0.37) and negative for IgG− (*ρ* > −0.48).

### 3.5. Assessment of Adherence for Tuberculosis Treatment and Adverse Reactions

**Figure 5:**
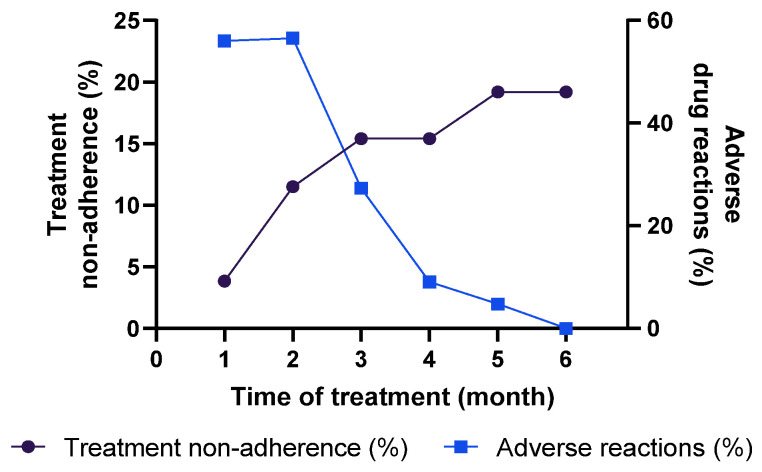
Assessment of treatment adherence and adverse drug reactions of individuals with active TB (*n* = 26).

The adherence to treatment was assessed monthly through phone calls. Among the 36 patients enrolled in this study, ten did not answer the calls and were excluded, resulting in 26 patients (16 positive for anti-SARS-CoV-2 IgG and 10 negative for anti-SARS-CoV-2 IgG) concluding the follow-up study. After the 1st month, one patient abandoned the treatment (treatment non-adherence: 3.85%), two in the 2nd month (11.5%), and another in the 3rd month (15.4%) (Figure 5). Another patient abandoned the treatment in the 5th month, and the final proportion of cases of noncompliance with treatment was 19.2%. Considering the hypothesis that a previous exposure to SARS-CoV-2 could result in more severe TB presentation and consequently negatively affect the adherence to treatment [20,21], we compared the final rate of treatment non-adherence in IgG+ and IgG- groups (23.5% and 10%, respectively). Statistical differences were not observed between these rates (*Χ*^2^ = 0.7638; *p* = 0.3821).

Regarding adverse drug reactions, approximately 70% of TB patients reported at least one adverse drug reaction during the treatment. The highest rates were reported in the 1st (56%) and 2nd (56.5%) months (Figure 5). Considering the patients with adverse reactions in the 1st and 2nd months, pruritis was the most prevalent side effect (42.9% and 46.2%, respectively), followed by nausea (35.7% and 30.8%) and weakness (14.3% and 15.4%). In the following months, side effects were reported by 27.3%, 9.1%, and 4.8% of TB subjects in the 3rd, 4th, 5th months, respectively. In these months, pruritis was also the most reported reaction (83.3–100%). Finally, in relation to the treatment outcome, all patients that completed the treatment (21/26; 80.7%) reported that the treatment was effective, and they did not have to start a new cycle of chemotherapy for tuberculosis. 

## 4. Discussion

TB is considered a serious public health problem worldwide, being responsible for the death of thousands of people [5]. Although TB is a priority for the Brazilian health system, several challenges are still being faced to improve the efficiency of disease control and reduce treatment abandonment [22]. This situation is estimated to have worsened during the COVID-19 pandemic due to the disruption of health services and other related complications [6,7]. In this context, the present study evaluated the clinical conditions and pre-therapy serum biomarkers of individuals who started the treatment for TB in São Luís during the COVID-19 pandemic, along with the treatment adherence.

Firstly, it was observed that most patients started the treatment based on clinical symptoms and radiological diagnosis. This conduct is in accordance with the recommendations of the Brazilian Thoracic Association, given the context of limited resources, and the benefits of early diagnosis for treatment efficiency and the reduction of the transmission [18,23,24]. Chest radiography is a method of significant importance as it is easy to perform, accessible, inexpensive, and safe (using a low dose of radiation). It should be requested for all patients with clinical suspicion of pulmonary TB [25]. Despite its low specificity, chest radiography is extremely valuable for physical examination, as it can detect multiple clinical changes and exclude associated lung disease [18]. On the other hand, chest computed tomography (CT) is more sensitive and specific than radiography in detecting early clinical changes, being able to distinguish active lesions from residual lesions in most cases. This method accurately assesses the duration of the disease and determines a diagnostic pattern based on the main changes observed [18].

It was also possible to observe that imaging methods were combining GeneXpert^®^ rapid molecular test and/or smear. The GeneXpert^®^ rapid molecular test, introduced in 2014 in Brazil, demonstrates some advantages, including speed, standardization, and sensibility, although conventional smear and culture methods continue to be considered the gold standard for diagnosis and follow-up of TB treatment [26]. Less than 40% of patients enrolled in this study were submitted to sputum smear evaluation. Sputum smear is a simple and safe method that, if correctly performed, allows the detection of 60% to 80% of cases of pulmonary TB in adults. This is a fundamental fact, considering that cases with a positive smear microscopy are the main ones responsible for the maintenance of the disease transmission chain [27]. Although Sputum culture remains the gold standard method for diagnosing TB, the patients enrolled in this study either started the treatment without the results or this exam was not solicited. Certainly, this occurred due to the time it takes to have the confirmation of the result (4 to 8 weeks) [28].

In the present study, most TB patients were males aged between 18 and 34 years (covering an economically active adult population) and declared low family income. Previous studies have linked these socioeconomic factors to the exposure of individuals to environments with greater circulation and contamination of the disease [29]. Regarding the signs and symptoms, cough, weight loss, fever, and weakness were the most prevalent symptoms. Cough is the earliest and most frequent symptom of pulmonary tuberculosis (usually accompanied by sputum) and is the mechanism of transmission [30]. However, the layperson does not associate these symptoms with the disease and rarely seeks a health unit (passive search) at the onset of symptoms that are initially attributed to flu-like symptoms, smoking bronchitis, or any other clinical situation [31].

During the COVID-19 pandemic, several co-infections with respiratory pathogens have been reported, including bacterial and fungal pathogens [32]. In this sense, seroprevalence studies focused on patients under TB treatment are important to provide insights into the dynamics of SARS-CoV-2 transmission in this population and to assist in the formulation of public strategies for these vulnerable people [33]. Indeed, the interactions between COVID-19 and TB have been evaluated; for instance, one study reported that of forty-nine patients, 18.3% were diagnosed with COVID-19 and TB in the same week, while 28.5% had COVID-19 before TB diagnosis and 53% were diagnosed with TB first [21]. In our study, we did not detect anti-SARS-CoV-2 IgM antibodies in TB individuals, while the prevalence of anti-SARS-CoV-2 IgG antibodies was 67%. This rate was superior to the seroprevalence of 26.5% reported for patients under TB treatment in Rio de Janeiro (Brazil) between September 2020 and February 2021 [33].

Despite the prevalence of anti-SARS-CoV-2 IgG in TB patients enrolled in the present study, only 16.7% of them reported being diagnosed with COVID-19 before TB diagnosis. It is important to highlight that the vaccine against SARS-CoV-2 was introduced in Brazil during the study period, which limited our serological study. Another important observation is that not all previously SARS-CoV-2-exposed individuals will test positive in serological assays as some factors could influence the results, such as time of testing after exposure/vaccination and type of test. In addition, other assays, such as qPCR and rapid SARS-CoV-2 antigen tests, would be more appropriate to detect co-infection.

All TB patients presented CRP levels higher than expected reference values and higher than comparison subjects. CRP is a non-specific acute-phase protein and a sensitive biomarker of inflammation, infection, and tissue damage. Elevated levels of CRP (alone or in combination with other markers) are indicative of bacterial and viral infections [34]. Elevated CRP levels in TB patients have been indicated as a risk factor for lung cancer [35]. Furthermore, CRP has been pointed out as an alternative test to be added to improve the diagnosis of TB in people living with HIV [15,36]. The CRP levels were significantly increased in TB patients without anti-SARS-CoV-2 IgG antibodies. It is important to highlight that CRP is one of the inflammatory biomarkers used to evaluate COVID-19 severity [37].

TB is one of the granulomatous diseases that alter the status of the plasma concentration of serum calcium [38]. While some studies report TB-associated hypercalcemia [39,40], others report hypocalcemia [41,42]. In the present study, most TB patients (88.9%) presented hypocalcemia, while 5.55% had hypercalcemia. The high prevalence of hypocalcemia is associated with malnutrition and TB-induced malabsorption. In addition to hypocalcemia, decreased plasma albumin and active vitamin D metabolites are commonly seen in TB patients [17,43]. Another crucial factor to be considered is that TB affects people living in disadvantaged conditions, such as dark houses with poor exposure to sunlight, which results in vitamin D deficiency and low nutritional intake leading to hypocalcemia [38,44]. The dyslipidemia observed in TB patients enrolled in our study also reflects their poor nutritional status. 

Hepatic function was evaluated in this study by measuring the levels of AST, ALT, GGT, and ALP. Many TB subjects presented elevated concentrations of ALP and GGT, indicating liver dysfunction before the treatment. In addition, TB patients had higher levels of all tested liver enzymes when compared with the comparison group. These results deserve attention since hepatotoxicity is one of the most frequent and serious adverse effects of anti-TB drugs [45,46]. Therefore, monitoring of liver function is necessary during treatment, especially in patients with a history of alcohol/drug consumption, previous and/or current liver disease, who are HIV-infected, or using other hepatotoxic medications [14].

Few TB patients (13.9%) showed changes in serum levels of amylase; however, this enzyme was significantly higher in TB patients than in comparison subjects. Comparable results were reported by Motswaledi et al. [47]; however, these authors included in their study patients that were under treatment; thus, they could not dismiss the possibility that the elevation in amylase activity was the result of anti-TB drugs. The role of amylase in TB pathogenesis remains unclear [47]. We also observed a higher amount of amylase in TB patients previously exposed to SARS-CoV-2 or vaccinated.

In relation to adherence to TB treatment during the analyzed period, the treatment was abandoned by 19.2%. This rate was similar to others observed in some studies performed before the pandemic period in Brazilian cities such as Fortaleza (2007–2014, 15.4%) [48], Rio de Janeiro (2008–2012, 13.8%) [49], Salvador (2014–2016, 17.6%) [50]. Adherence among TB patients is a challenge due to several factors, including treatment time, side effects, the distance between home and the health center, drug consumption, and TB symptom improvement at the beginning of treatment [10,13]. In this sense, a large study with a greater number of patients should be performed to better characterize the impact of COVID-19-related restrictions on adherence of TB treatment.

## 5. Conclusions

The present study evaluated the clinical features and biochemical markers of 36 individuals who started the treatment for TB in São Luís during the COVID-19 pandemic. The condition was predominant in males, those of economically active age, and with low family income. It was observed that the treatment started in most of the cases only based on image methods, even though the molecular assay was available in the hospital. This finding indicates the need for actions to adjust the routine of this service.

Before starting the treatment, these patients showed elevated levels of CRP, low levels of HDL, and hypocalcemia. Liver and pancreatic functions were also compromised in several patients. Taken together, these data point out the importance of including the evaluation of serum markers as complementary exams in the routine of TB diagnosis, which is not a reality in the studied hospital. In addition, the patients were followed up for six months, and the rate of non-adherence to treatment was 19.2%. 

Some limitations are found in the present study—for instance, the lack of Septum culture and Xpert MTB/RIF for some patients, which could result in the inclusion of individuals without TB in the group under treatment. However, all patients were diagnosed using the recommendations of the Brazilian Thoracic Association, considering the COVID-19 period and situations of limited resources. Another important factor is that the patients receive the drugs for treatment in different primary health services throughout the region and do not return to the hospital. This makes it difficult to contact the patients, and the use of phone calls to monitor treatment adherence and outcome, even with the associated limitations, was the only resource. In this sense, a large study monitoring the serum biomarkers during and after the treatment would be interesting. Despite these limitations, this report presents an important description of the reality faced by the service during the pandemic situation. Furthermore, it provides more insights into the value of serum biomarkers in TB patients, in particular, CRP, calcium, liver and pancreatic enzymes.

## Figures and Tables

**Table 1:** Sociodemographic characteristics of individuals recently diagnosed with active pulmonary tuberculosis and the comparison group.

Variables	TB Patients *n* (%)	Comparison Group *n* (%)
**Gender**		
Male	25 (69.4)	24 (68.6)
Female	11 (30.6)	11 (31.4)
**Age (years)**		
18–24	08 (22.2)	09 (25)
25–34	11 (30.5)	11 (30.5)
35–44	07 (19.4)	07 (19.4)
45–54	06 (16.6)	06 (16.6)
55–64	03 (8.3)	02 (5.5)
65+	01 (3.0)	01 (3.0)
**Marital status**		
Single	20 (55.6)	24 (68.6)
Married	16 (44.4)	11 (31.4)
**Education level**		
No formal education	7 (19.4%)	1 (2.9)
Elementary School	6 (16.6%)	2 (5.7)
High School	20 (55.7%)	21 (60.0)
Higher Education	3 (8.3%)	06 (17.1)
Postgraduate studies	-	05 (14.3)
**Ethnic-racial identity**		
White	2 (5.6)	09 (25.7)
Pardos (mixed)	24 (66.7)	21 (60.0)
Black	10 (27.8)	05 (14.3)
**Family income**		
Up to 3 minimum wages *	33 (91.7)	22 (62.9)
More than 3 minimum wages *	3 (8.3)	13 (37.1)

* Approximately 600 USD.

**Table 2:** Additional health characteristics of individuals recently diagnosed with active pulmonary tuberculosis and the comparison group.

Characteristics	TB Patients	Comparison Group
	*n* (%)	*n* (%)
Contact with people with Tuberculosis	18 (50.0)	7 (20.0)
Contact with individuals with Leprosy	4 (11.1)	4 (11.4)
Presence of the vaccine scar	29 (80.6)	31 (88.6)
Tuberculin test		
Reactor	7 (19.4)	0
No reactor	0	1 (2.9)
Unrealized	28 (77.8)	34 (97.1)
Comorbidities	6 (16.8)	3 (8.6)
Other lung diseases	3 (8.4)	0
Self-reported Smoking	11 (30.6)	-
Frequent use of alcohol	17 (47.2)	-

(-) Data not collected.

**Table 3:** Prevalence of anti-SARS-CoV-2 antibodies, COVID-19 symptoms, and vaccination status of newly-diagnosed tuberculosis patients and the comparison group.

	TB Patients		Comparison Group
	IgG (−) *n* (%)	IgG (+) *n* (%)	IgG (+) *n* (%)
Prevalence of anti-SARS-CoV-2 IgG	12 (33.3)	24 (66.7)	35 (100)
Previous diagnosis for COVID-19	0	6 (25)	14 (40)
Typical symptoms of COVID-19	3 (25)	12 (50)	16 (45.7)
Contact with subjects with COVID-19	4 (33.3)	11 (45.8)	22 (62.8)
Vaccination for COVID-19	2 (16.7)	11 (45.8)	35 (100)
Astrazeneca	0	8 (33.3)	19 (54.3)
Coronavac	1 (8.3)	0	13 (37.1)
Pfizer	0	3 (12.5)	9 (25)
Do not know	1 (8.3)	0	0

**Table 4:** Clinical features in newly-diagnosed tuberculosis patients and the correlation with COVID-19 serology.

Signal and Symptoms	TB Patients			*Χ* ^2^	*p*
	Total	Anti-SARS-CoV-2			
		IgG (−)	IgG (+)		
Cough	33 (91.7)	11 (87.5)	24 (100)	1.636	0.2008
Weight loss	32 (88.9)	10 (83.3)	24 (100)	2.250	0.1336
Fever	30 (83.3)	9 (75)	24 (100)	3.600	0.0578
Weakness	21 (58.3)	5 (45.8)	20 (83.3)	4.629	**0.0314**
Inappetence	10 (27)	3 (25)	8 (33.3)	0.2769	0.5987
Hemoptysis	8 (22.2)	2 (20.1)	6 (25)	0.2835	0.7768

**Table 5:** Biochemical alterations observed in newly-diagnosed tuberculosis patients before the treatment initiation.

Abnormalities	*n*	%
Low HDL (M: <55 mg/dL; F: <65 mg/dL)	36	100
High CRP (>6 mg/dL)	36	100
Hypocalcemia (<8.8 mg/dL)	32	88.9
High ALP (>100 U/L)	17	47.2
High GGT (M: >55 U/L; F: >38 U/L)	17	47.2
High Triglycerides (>120 mg/dL)	8	22.2
High Amylase (>125 U/L)	5	13.9
High Uric acid (M: >7 mg/dL; F: >6 mg/dL)	3	8.3
High ALT (M: >39 U/L; F: >37 U/L)	2	5.6
High AST (M: >45 U/L; F: >37 U/L)	2	5.6
Hypercalcemia (>11 mg/dL)	2	5.6
High Total Cholesterol (>120 mg/dL)	1	2.8

## Data Availability

Data available upon request.

## Supplementary Materials

The following supporting information can be downloaded at: https://www.mdpi.com/article/10.3390/jcm12144843/s1, Table S1: Follow-up of adherence to treatment of pulmonarytuberculosis cases.
