# Supplementary material for: Clinical Features, Biochemical Parameters, and Treatment Adherence of Individuals Who Started the Treatment for Active Pulmonary Tuberculosis during the Pandemic Period
Source: J Clin Med. 2023 Jul 22;12(14):4843. doi: 10.3390/jcm12144843 (PMC10381187; doi:10.3390/jcm12144843)
Supplement: Supplementary file 1 [file jcm-12-04843-s001.zip › jcm-2085668-supplementary.pdf]

Table S1

FOLLOW-UP OF ADHERENCE TO TREATMENT OF PULMONARY TUBERCULOSIS CASES

Name:_______________________________________

Month of treatment: (   ) 1º (   )2º (   )3º (   )4º (   )5º (   )6º

Are you following the prescribed treatment?

Treatment phase:

(   ) Intensive phase (2 months)   (   ) Maintenance phase (4 months)

Has the treatment regimen changed?

(   ) Yes   (   ) No   Why? _______________________________________

Did you have any adverse reaction to the medication used in the treatment this month?

(   ) Yes   (   ) No   Which? _______________________________________
